# Bta-miR-124a Affects Lipid Metabolism by Regulating* PECR* Gene

**DOI:** 10.1155/2019/2596914

**Published:** 2019-07-28

**Authors:** Binglei Shen, Zhuonina Yang, Shuo Han, Ziwen Zou, Juan Liu, Lian Nie, Wentao Dong, E. Li, Shengjun Liu, Zhihui Zhao, Rui Wu

**Affiliations:** ^1^College of Animal Science and Veterinary Medicine, Heilongjiang Bayi Agricultural University, 163319, Heilongjiang Daqing, China; ^2^Heilongjiang Provincial Key Laboratory of Prevention and Control of Bovine Diseases, 163319, Heilongjiang, China; ^3^College of Agricultural, Guangdong Ocean University, 524088, Zhanjiang, China

## Abstract

According to our previous studies, bta-miR-124a was differentially expressed in breast tissue between high-fat and low-fat dairy cows. However, the function of bta-miR-124a in lipid metabolism of dairy cows and the identification of its target genes have not been reported. Therefore, this study will identify the target gene of bta-miR-124a and explore its role in the regulation of milk lipid metabolism. First, preliminary bioinformatics prediction of bta-miR-124a candidate target genes was performed, and quantitative real-time polymerase chain reaction (qRT-PCR) was used to analyze relative expression changes of bta-miR-124a and its candidate target genes and the expression level of the downstream gene of the target gene in the lipid metabolism signaling pathway in dairy mammary epithelial cell lines (Mac-T), using the dual luciferase reporter system for the identification of the targeting relationship between bta-miR-124a and the candidate target gene. Then, the effect of transfection of bta-miR-124a mimics and inhibitors on triglyceride (TG) and free fatty acid (FFA) levels was analyzed. The results indicate that bta-miR-124a directly interacts with the 3′-untranslated region of peroxisomal trans-2-enoyl-CoA reductase (*PECR*) to downregulate its expression in Mac-T cells. Further, bta-mir-124a regulates the expression of* PECR* and the downstream gene extension of very long chain fatty acid protein 2 (*ELOVL2*) through an unsaturated fatty acid biosynthesis signaling pathway. In conclusion, bta-miR-124a is involved in lipid metabolism by directly downregulating the* PECR* gene and affecting the expression of the downstream gene* ELOVL2* and regulates the content of some key secretory elements such as TG and FFA. The function of bta-miR-124a has a certain effect on the synthesis and secretion of milk fat in the mammary epithelial cells of dairy cows.

## 1. Introduction

As people's dietary needs increase, consumers are increasingly paying attention to the fat content of dairy products, which has prompted livestock farmers to produce more diverse dairy products to meet consumer demand. Milk fat is an important nutrient component of milk and a major indicator used to measure milk quality in dairy production.

MiRNAs are noncoding small RNAs of about 20 nucleotides in length that mediate posttranscriptional regulation by forming RNA-induced silencing complexes with related proteins, thereby playing an important role in cell differentiation and proliferation [1–3].

Numerous studies have confirmed that many microRNAs (miRNAs) have an effect on milk fat metabolism in dairy cows. Lipid metabolism-related miRNAs have been reported to be involved in lipid synthesis, transport, and oxidation to regulate lipid metabolism [4, 5]. The miRNAs and their target genes are cross-regulated, and such regulatory features lead to the complexity of lipid metabolism regulation involved in miRNAs [6]. At present, some studies have shown that miRNAs can participate in the development of breast, proliferation, and differentiation of breast cells, milk fat synthesis, lactation, and other processes. This strongly suggests the importance of miRNAs in milk fat metabolism.

Our previous studies have identified several lipid metabolism-related miRNAs. Bta-miR-224 could affect apoptosis and triglyceride (TG) synthesis by regulating acyl-CoA dehydrogenase medium chain (*ACADM*) and aldehyde dehydrogenase 2 (*ALDH2*), which are two important genes involved in lipid metabolism in Mac-T cells [7]. In another study, bta-miR-33a negatively regulated the expression of elongation of very long chain fatty acids protein 5 (*ELOVL5*), elongation of very long chain fatty acids protein 6 (*ELOVL6*), and sterol-C4-methyl oxidase (*SC4MOL*) in mammary gland with low milk fat content (MG-LL); bta-miR-152 was downregulated in MG-LL and the significantly upregulated genes were prostaglandin-endoperoxide synthase 2 (*PTGS2*), protein kinase AMP-activated catalytic subunit alpha 1 (*PRKAA1*), and uncoupling protein 3 (*UCP3*) [8]. In addition, another research reported that the overexpression of miR-130a significantly decreased cellular triacylglycerol (TG) expression level and suppressed lipid droplet formation, whereas the inhibition of miR-130a resulted in greater lipid droplet formation and TG accumulation in bovine mammary epithelial cells [9].

MiR-124 is a small RNA molecule located on chromosome 8. After comparing the precursor sequence and mature sequence of miR-124, we found that the miR-124 sequence is highly conserved among species. MiR-124 has been studied for many years; most studies were related to brain and neurons development. Researchers found that the tumor suppressor activity of miR-124 could be partly due to its inhibitory effects on glioma stem-like traits and invasiveness through Snail homolog 2 (*SNAI2*) [10]. Silber et al. reported that miR-124 inhibit proliferation of glioblastoma multiforme cells and induce differentiation of brain tumor stem cells [11]. Eugene V et al. reported that miR-124 promotes neuronal differentiation by triggering brain-specific alternative pre-mRNA splicing [12].

In recent years, miR-124 has also been shown to be involved in lipid metabolism and affect lipid synthesis. Das et al. reported that miR-124a attenuates RNA and protein expression of the major TG hydrolase, adipose TG lipase (*ATGL/PNPLA2*), and its coactivator comparative gene identification 58 (*CGI-58/ABHD5*) [13]. Holmes et al. found miR-124/506 and miR-108 binding sites in the 3′-noncoding region of mRNA, including peroxisome proliferator-activated receptor alpha (*PPARA*) and peroxisome proliferative activated receptor gamma (*PPARG*), which are key regulators of genes encoding enzymes of lipid metabolism [14]. Those provided a basis for the study of miR-124a in lipid metabolism and also indicated that miR-124a plays an important role in fatty acid metabolism. However, the study of miR-124a involved in fatty acid metabolism in dairy cows is limited. We had reported that bta-miR-124a was differentially expressed between high-fat and low-fat dairy cow primary mammary epithelial cells with high significance [8]. Therefore, this experiment attempted to identify the target gene of bta-miR-124a and explore its effect on lipid metabolism in dairy cows, in order to provide a basic reference for molecular-oriented breeding of dairy cows.

Peroxisomal trans-2-enoyl-CoA reductase (*PECR*) is also called TERP and TECR, which is located on chromosome q35 (chr2:215996329-216082955) with 86627 bases (https://www.genecards.org/). It can participate in carbon chain elongation in fatty acid metabolism and catalyze the last reaction of four long chain fatty acid elongation cycles. Each cycle of* PECR* adds two carbons to the long chain and very long chain fatty acid (VLCFA) chains, reducing the intermediate of trans-2,3-enoyl coenzyme A fatty acid to acyl coenzyme A, which can further extend the carbon chain by entering a new elongation cycle [15, 16]. Meanwhile,* PECR *is a peroxisome protein involved in fatty acid synthesis and plays an important role in milk fat synthesis. Studies have shown that the gene is closely related to the late development of TG [17]. Therefore, in this study, we reported the expression of bta-miR-124a in Mac-T cells, which can affect lipid metabolism by regulating* PECR* gene, which provided a reference for further study on the regulation mechanism of milk fat in dairy cows.

## 2. Materials and Methods

### 2.1. Bioinformatics Prediction of Candidate Target Genes of bta-miR-124a

The mature sequences of bta-miR-124a were obtained on miRBase (http://www.mirbase.org/index.shtml) and homology analysis was performed. The possible target genes for bta-miR-124a are predicted by TargetScan (http://www.targetscan.org). The bioinformatics website Starbase (http://starbase.sysu.edu.cn/) was used to predict the extent of nonspecific pairing of bta-miR-124a with its candidate target genes.

### 2.2. Cells Culture and Transfection

Mac-T cells were from the laboratory in Heilongjiang Bayi Agricultural University. Mac-T cells were cultured in Dulbecco's modified Eagle's medium and F-12 (DMEM/F12) (Gibco, Grand Island, NY) with 10% fetal bovine serum (FBS) (Gibco, Grand Island, NY, USA) 100 U/mL penicillin, and 100 *μ*g/mL streptomycin with or without 250 ng/mL amphotericin B (Invitrogen, Carlsbad, CA, USA) digestive cells with 0.25% trypsin (Solarbio, Beijing, China) and incubated at 37°C in a humidified atmosphere of 5% CO_2_.

The indicated cells reaching 60% to 80% confluence were transfected with 10nM of bta-miR-124a mimics or inhibitors and their corresponding control group constructs (Mir-shNC, Anti-NC) (Sangon Biotech, Shanghai, China) in 6-well using Lipofectamine 3000 (Invitrogen Life Technologies, Carlsbad, CA, USA). The medium was changed to regular cell culture medium after 4-6 h. Cells were collected for analysis at 24-48 h after transfection.

### 2.3. Total RNA Isolation and Quantitative Polymerase Chain Reaction (qPCR) Assays

Forty-eight hours after transfection with the miR-124a mimics/inhibitors, total RNA of the Mac-T cells was extracted using trizol reagent (Invitrogen). The quantity of RNA was verified by NanoDrop1000 (Thermo Scientific), ensuring the optical density (OD) 260nm/280nm value was between 1.8 and 2.0. Complementary DNA (cDNA) was synthesized using the Primer Script reverse transcription PCR (RT-PCR) kit (Takara). The expressions of miR-124a and target genes were detected on a Bio-Rad CFX Connect instrument (Biorad, America) using the SYBR Green I (TOYOBO, Japan). Primer 6.0 (Premier) was used to design fluorescently labeled primers and reverse transcription primers for quantitative analysis of miR-124a,* PECR*, and* ELOVL2* (enhanced extension of very long chain fatty acid protein 2). The expression of* U6* and* GADPH* (glyceraldehyde-3-phosphate dehydrogenase) was used as a loading control. All primers were synthesized by Shanghai Sangon Biotechnology Company (see Table 1). The relative gene expression was calculated by the 2^−ΔΔCt^ method.

### 2.4. Vectors Construction

Vectors construction of miR-124a mimics, miR-124a inhibitors, and corresponding control group (Mir-NC, Anti-NC) were purchased from Shanghai Sangon Biotech Company. The* PECR*-mut/WT/si vectors were purchased from GenePharma Company.

### 2.5. Luciferase Activity Assay

Mac-T cells were cultured in 6-well plates the day prior to transfection. All transfections were carried out with Lipofectamine 3000 (Invitrogen). The PECR mut/WT/si vectors were cotransfected with bta-miR-124a into Mac-T cells. After 48 h of transfection, luciferase activities were measured using the Dual-Luciferase Reporter Assay System (Promega) and normalized to renilla luciferase activity. The experiments were performed in triplicate.

### 2.6. Detection of TG and FFA Detection Content

After 48 h of transfection, cultured cells were collected. The expression levels of TG and FFA in Mac-T cells were assayed using the cell TG assay kit and FFA assay kit (Jiancheng, Nanjing, China). All of the above experiments were performed according to the manufacturer's recommended protocol.

### 2.7. Statistical Analysis

The results were analyzed using Random Design of Single Factor Analysis of Variance between groups based on SPSS 22.0 software. All results were presented as the means ± standard error of mean (SEM) of separate experiments (n >=3). Statistical significance is presented as *∗p*< 0.05, *∗∗p*< 0.01.

## 3. Results

### 3.1. Sequence Homology Analysis of Bta-miR-124

**Figure 1:**
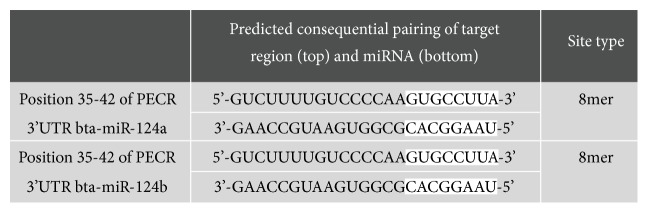
Predicted binding sites of bta-miR-124a on 3′-untranslated region (3′-UTR) of peroxisomal trans-2-enoyl-CoA reductase (*PECR*).

In the miRBase database, the bta-miR-124a base sequence was obtained, and the mature sequence of bta-miR-124 was found to be highly conserved among species. Using the predictive software TargetScan 7.2, results showed that the miR-124a target site is present at 36-43 nt of the* PECR* 3′ UTR (see Figure 1). Based on above findings, the regulatory relationship analysis of bta-miR-124a and its candidate target gene* PECR* was performed in Mac-T cells.

### 3.2. Bta-miR-124a Downregulated Its Candidate Target Gene* PECR*

**Figure 2:**
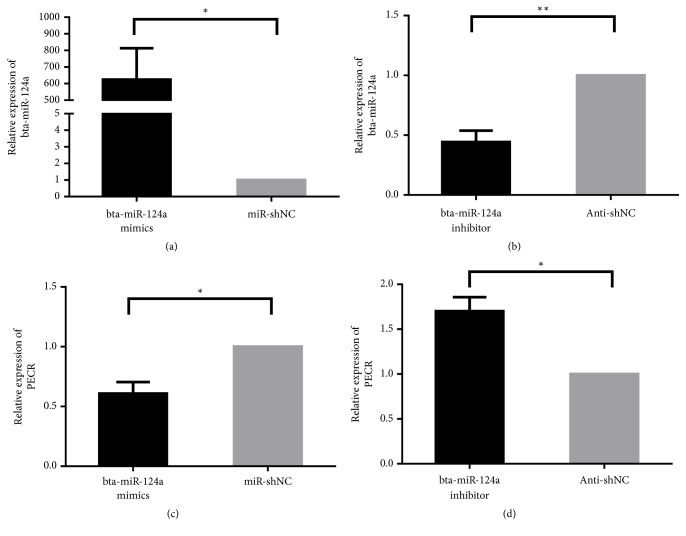
Expression levels of bta-miR-124a and* PECR* after transient transfection with bta-miR-124a mimics/inhibitors in Mac-T cells. (a, b) The expressions of bta-miR-124a in Mac-T cells that transfected bta-miR-124a mimics, bta-miR-124a inhibitors, control group (Mir-shNC and Anti-NC) (*∗p *<0.05, *∗∗p *<0.01). (c, d) The mRNA expressions of* PECR* gene in Mac-T cells that transfected bta-miR-124a mimics, bta-miR-124a inhibitors, and control group (Mir-shNC and Anti-NC) (*∗p* <0.05).

**Figure 3:**
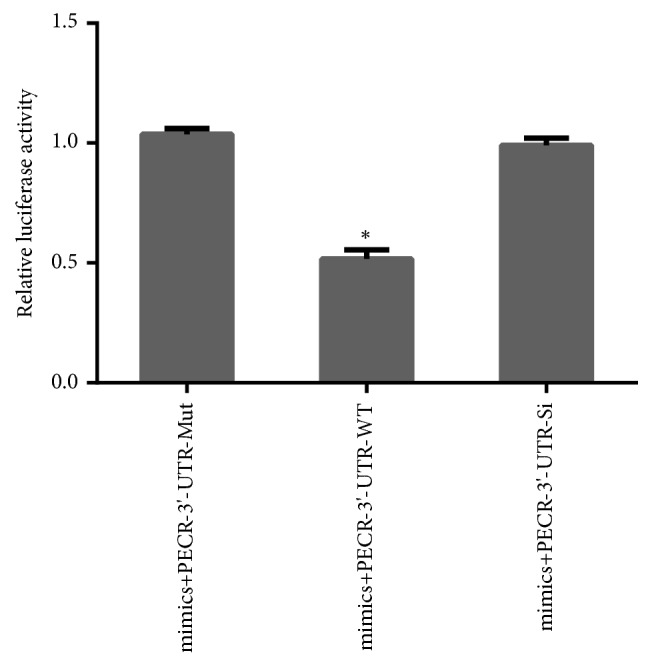
Luciferase activity assay report target relationship. Luciferase activities were detected in Mac-T cells that miR-124a cotransfected with* PECR*-mut/WT/NC vector (*∗p* <0.05).

After transfection with bta-miR-124a mimics, inhibitors, and control group (Mir-shNC and Anti-NC) into Mac-T cells, respectively, the corresponding expression levels were detected by quantitative real-time PCR (qPCR) whose primer sequence was designed by primer 6.0. The results showed that the expression of bta-miR-124a mimics was significantly higher than that of the control group, and the bta-miR-124a inhibitors were lower than the control group (see Figures 2(a) and 2(b)). Compared with the control group, the expression of* PECR* gene decreased after the transfection of bta-miR-124a mimics (see Figure 2(c)) and increased slightly after the transfection of bta-miR-124a inhibitors (see Figure 2(d)). Furthermore, the 3′-UTR of wild-type* PECR* was linked to pmirGlo (Promega) and examined in Mac-T cells that had been transfected with bta-miR-124a mimics or a control group. A significant decrease in reporter activity was observed when using the bta-miR-124a mimics transfected with wild type 3′ UTR-reporter of* PECR* compared to the control. The transfection with bta-miR-124a mimics had little effect on the luciferase activity of the mutant 3′ UTR-reporter of* PECR* compared with the control (see Figure 3). The above results indicated bta-miR-124a could directly target* PECR* gene.

### 3.3. Bta-miR-124a Regulates Downstream Genes of* PECR*

**Figure 4:**
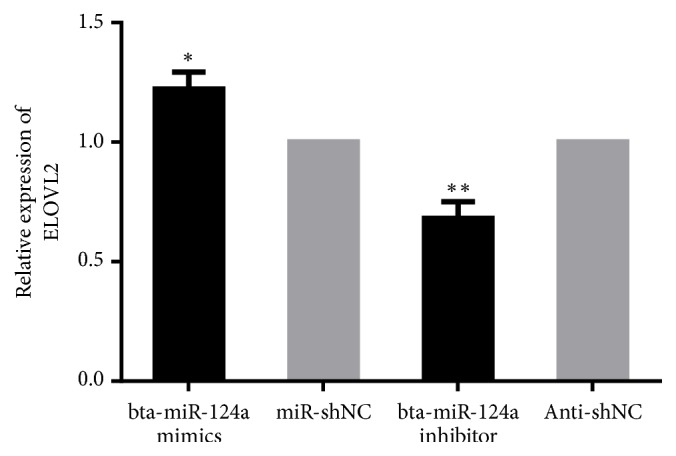
Expression of* ELOVL2* after the overexpression and inhibition of bta-miR-124a in Mac-T cells: mRNA level of* ELOVL2* after treatment with bta-miR-124a mimics and control group (miR-NC) in Mac-T cells. mRNA level of* ELOVL2* during treatment with bta-miR-124a inhibitors and control group (Anti-NC) in Mac-T cells (*∗p *<0.05, *∗∗p *<0.01).

The interaction between miRNAs and target genes may affect a series of downstream genes. Therefore, we obtained the lipid metabolism signal transduction pathway involved in* PECR* from the KEGG database. The* PECR *downstream gene* ELOVL2 *was observed in the lipid metabolism pathway. After transfection of bta-miR-124a mimics and inhibitors and their control group, it was noticed that the expression of* ELOVL2* increased after transfection by bta-miR-124a mimics (see Figure 4), and the expression of* ELOVL2* decreased after transfection by bta-miR-124a inhibitors (see Figure 4). The above results suggest that bta-miR-124a plays an important role in the biosynthesis of unsaturated fatty acids signaling pathway.

### 3.4. Overexpression of Bta-miR-124a Upregulates TG and FFA Levels

**Figure 5:**
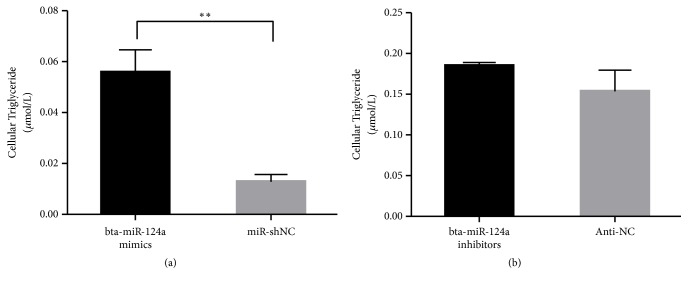
The cellular triglyceride in Mac-T cells that transfected bta-miR-124a mimics, bta-miR-124a inhibitors, and control group (Mir-shNC and Anti-NC) (*∗∗p *<0.01).

**Figure 6:**
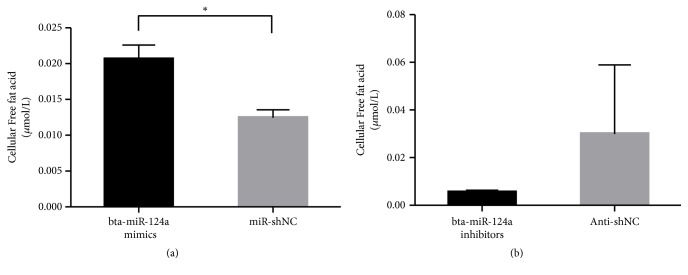
The cellular free fat acids content in Mac-T cells that transfected bta-miR-124a mimics, bta-miR-124a inhibitors, and control group (Mir-shNC and Anti-NC) (*∗p *<0.05).

The synthesis and secretion of lipids in mammary epithelial cells are regulated by lipid metabolism-related genes. Therefore, the contents of TG and FFA in Mac-T cells were examined after the overexpression and inhibition of bta-miR-124a. Compared with the control group, the content of TG and FFA in Mac-T cells transfected with mimics was increased, and the difference was significantly (*∗∗p *<0.01). The content of TG and FFA in the transfected inhibitors group was decreased compared with the control group as well, but there was no significant difference in TG content (see Figures 5 and 6).

## 4. Discussion

Lipid metabolism is one of the three major nutrients metabolisms, which is mainly involved in the energy supply and storage of the body, the composition of biofilm, and its function. As a regulator of lipid metabolism and lactation in dairy cows, miRNAs play an important role in the growth and development of dairy cows and milk production. It has been confirmed that miR-33, miR-548p, and miR-30c are mainly involved in lipid synthesis and transport process, while miR-122 and miR-370 are mainly involved in lipid synthesis and oxidation utilization [18–23]. In Chinese Holstein cows, research has been reported that bta-miR-181a regulates the biosynthesis of bovine milk fat by targeting acyl-CoA synthetase long chain family member 1 (*ACSL1*) [24]. Bta-miR-130a regulates the biosynthesis of bovine milk fat by targeting peroxisome proliferator-activated receptor gamma [9]. Das et al. have shown that ectopic expression of miR-124a in adipocytes can lead to decreased fat decomposition and increased cell TG accumulation [25].

According to previous research, miR-124a was dominantly shown to be a potential prognostic factor for breast cancer, myelodysplastic syndrome, and prostate cancer [26, 27]. Meanwhile, miR-124a regulates the expression of multiple genes. miR-124a is particularly highly expressed in differentiated and mature neurons, but is lowly in neural stem cells, neural precursor cells and glial cells. It is also the most expressed miRNAs in the mammalian nervous system, accounting for 50.48% of the total miRNA in the mammalian cerebral cortex [11]. In recent years, the research of miR-124a was not limited to the brain but also involved in the lipid metabolism process. Research showed that the 3′-noncoding regions of human* ATGL* gene contains miR-124 binding sites, including transcription factor binding sites, such as* PPARA* and* PPARG*, which are key regulatory factors for encoding lipid metabolism enzyme genes [14]. Pan et al. reported that miR-124-3p inhibit adipogenesis by targeting branched chain keto acid dehydrogenase E1, alpha polypeptide (*BCKDHA*) mRNA, which not only revealed the role of miR-124-3p in regulating intramuscular fat formation but also provided a reference for understanding the role of* BCKDHA* in ovine adipogenesis [28]. However, dairy lipid metabolism has obvious difference from total lipid metabolism; the regulation function of miR-124 in dairy lipid for dairy cow is still limited.

Our previous study revealed that bta-miR-124a was differentially expressed between high-fat and low-fat dairy cow primary mammary epithelial cells with high significance [8]. Therefore, miR-124a may be a potential regulator of milk fat synthesis in dairy cows. However, the detailed functional mechanism is still unknown. In this experiment, the candidate target gene* PECR* was quantitatively analyzed by fluorescence, we found that the overexpression or silencing of bta-miR-124a in dairy cow resulted in downregulation or upregulation of the* PECR* gene, which suggest that bta-miR-124a can target the 3′UTR of* PECR* gene to regulate its expression. At the same time,* PECR* gene is involved in fatty acid metabolism pathway, suggesting that bta-miR-124a may indirectly affect the synthesis of milk fat by* PECR *gene.

*PECR* is a peroxisomal NADPH-specific trans-2-enoyl-CoA reductase that catalyzes the reduction of trans-2-enoyl-CoAs of varying chain lengths from 6:1 to 16:1, having maximum activity with 10:1 CoA. It plays a critical role in fatty acid elongation via the conversion of 2E-Octadecenoyl-coA to stearoyl-CoA [29]. It has been shown that* PECR* is expressed in CD4/CD8 T cells, B cells, dendritic cells, endothelial cells, smooth muscle, kidneys, liver, and adipocytes [30]. Dinavahi et al. reported that* PECR* plays an important role in the late synthesis of TG and in the treatment of fatty diseases [15]. At present, studies have shown that miRNA can participate in fatty acid metabolism, biosynthesis of unsaturated fatty acids, and peroxisomes by regulated of* PECR*. In 2015, Huang HY et al. studied the downregulation of* PECR* gene in chicken lipid metabolism by gga-miR-125b-3p, gga-miR-130b-3p, and gga-miR-26a-5p [31].

For the synthetic process of cow's milk fat is significantly different from other animals. The precursor of dairy cow milk fat synthesis is not mainly derived from the glucose produced by the decomposition of starch, but through the rumen fermentation, the final product of cellulose, hemicellulose and lignin, the final product of acetic acid, butyric acid, through the rumen wall Β-hydroxybutyric acid formed by oxidation of raw processes. Furthermore, mammary epithelial cells use *β*-hydroxybutyric acid as a precursor to synthesize fatty acids, eventually producing TG [32]. Acetic acid is mainly converted to acetyl-CoA by means of acetyl-CoA synthetase, and then acetyl-CoA is converted to malonyl-CoA by carboxylation. This step is the rate-limiting step of milk fat synthesis [33]. Fatty acid uses malonyl-CoA as a substrate to undergo four cycles of dehydrogenation, hydration, dehydrogenation, and thioester lysis. The carbon chain is gradually extended to synthesize the two main fatty acids in the body, stearic acid and palmitic acid. And stearic acid, other fatty acids, on the basis of C16 and C18, are extended and desaturated. Afterwards, we hypothesized that bta-miR-124a could affect the expression of the* ELOVL2* gene downstream of* PECR* in the lipid metabolism signaling pathway.* ELOVL2 *gene is a protein-coding gene and the main gene of long chain fatty acid family. It can participate in the metabolism of linolenic acid and the prolongation of fatty acids and is one of the important genes in lipid metabolism. As a downstream gene of* PECR*, it can extend the carbon chain with* PECR* gene, thus forming long chain fatty acids. To further identify the function of bta-miR-124a on the effect of lipid metabolism, the expression change of* ELOVL2* gene was detected. The results showed that the expression of* ELOVL2* was significantly increased after transfection of bta-miR-124a mimics and significantly decreased after transfection of bta-miR-124a inhibitors. The* PECR* gene is involved in the elongation of the carbon chain in fatty acid metabolism. Each cycle of* PECR* adds 2 carbons to the long chain and very long chain fatty acid chains and reduces the trans-2,3-enoyl-CoA fatty acid intermediate to acyl-Coenzyme A [15, 16]. By entering a new extended cycle and regulating the expression of* ELOVL2*,* ELOVL2* has been shown to be involved in C20 and C22 polyunsaturated fatty acid extensions. In fact, our study confirmed that* ELOVL2* expression changes in response to* PECR*, and* PECR* expression appears to be dependent on bta-miR-124a levels. Overexpression of bta-miR-124a inhibits the expression of* PECR* at the mRNA, thereby increasing the expression level of* ELOVL2*. The lipid metabolism process is a complex process in which PECR can participate in multiple carbon chain extensions in the lipid metabolism pathway. In the present study, when bta-miR-124a was overexpressed, the* PECR* of the trans-2,3-enoyl CoA fatty acid intermediate decreased and* ELOVL2* accumulated. When bta-miR-124a is inhibited, the expression of* PECR* is increased and the amount of* ELOVL2* is decreased. This may be due to the fact that the lipid metabolism process is often the result of multiple factors, indicating that* ELOVL2* is not only a downstream gene of* PECR*, but also regulated by other factors, so when* PECR* is inhibited,* ELOVL2* expression can be activated by other pathways.

In addition, we examined TG levels in mammary epithelial cells of dairy cows after the overexpression and inhibition of bta-miR-124a. The results showed that compared with the control group, the content of TG in transfected cells increased significantly (*p* <0.01). Compared with the control group, the content of TG in the transfection inhibitor group decreased, but there was no significant difference (*p* <0.05). Bta-miR-124a mimics are mature double strand of chemically synthesized miRNAs, which can enhance the function of endogenous miRNAs. The bta-miR-124a inhibitors are chemically synthesized methoxy-modified mature miRNA complementary single-strands, which specifically target inhibitors of specific target miRNAs in cells, and can effectively inhibit the activity of endogenous miRNAs in vivo. We speculate that the formation of TG may be affected only when the expression of bta-miR-124a is high. Further analysis, after inhibiting bta-miR-124a, there were significant differences in the level of target gene's mRNA, which proved that bta-miR-124a had a regulatory effect on target gene. However, the content of TG is not obvious, which may be due to the regulation of TG by many genes. In addition to the* PECR* gene, our undetected genes are involved in the regulation of TG. Overexpression of bta-miR-124a can inhibit the production of multiple target genes. Because of a dose compensation effect in the body, genes of TG synthesis pathway in cells actively mobilize to reach the original target gene level and reduce the impact of bta-miR-124a. At the same time, after the detection of free fatty acids, it was found that inhibition of bta-miR-124a could reduce the content of intracellular FFA, suggesting that bta-miR-124a may regulate lipid transport in the transport process but had no significant effect on the synthesis of TG.

FFA is mainly decomposed from neutral fats. Generally, single-stomach animals cannot absorb and digest FFA well, but cows as ruminants have evolved efficient fatty acid metabolism mechanism. During the feeding process of dairy cows, unsaturated fatty acids in roughage are hydrolyzed to FFA through rumen. At the same time, TG or a small portion of full-length chain fatty acids in feed are converted into FFA before absorption, which can be absorbed in the small intestine. As a kind of fatty acid, FFA can also affect the lipid synthesis of dairy cows through the mechanism of fatty acid metabolism. As a small biological molecule, miRNAs can indirectly regulate the secretion of FFA and can also be affected by FFA. In this study, we detected the level of FFA in mammary epithelial cells of dairy cows after transfection. The results showed that the content of FFA in mammary epithelial cells increased after the overexpression of bta-miR-124a, and the difference was significant (*p* < 0.05). Compared with the control group, the FFA in the bta-miR-124a inhibitor group decreased, with no significant difference (*p* < 0.05). According to the results of intracellular TG analysis, since FFA are the intermediate products of TG metabolism in vivo, there is a close relationship between FFA and TG. Bta-miR-124a plays an important role in regulating the content of TG and FFA. When bta-miR-124a was overexpressed, the metabolism of TG increased and the content of FFA increased. When bta-miR-124a was inhibited, the content of TG did not change significantly, but the content of FFA decreased. This study shows that bta-miR-124a can participate in the transport of lipids, affecting the transformation of intracellular TG and fatty acids, thereby affecting the production of milk fat.

In conclusion, bta-miR-124a is involved in lipid metabolism by directly downregulating the* PECR* gene and affecting the expression of the downstream gene* ELOVL2* and regulates the content of some key secretory elements such as TG and FFA.

## 5. Conclusion

In conclusion, bta-miR-124a is involved in lipid metabolism by directly downregulating the* PECR* gene and affecting the expression of the downstream gene* ELOVL2* and regulates the content of some key secretory elements such as TG and FFA. The function of bta-miR-124a has a certain effect on the synthesis and secretion of milk fat in the mammary epithelial cells of dairy cows.

## Figures and Tables

**Table 1:** Primer sequences of genes for quantitative real-time PCR.

Symbol	Primer Sequence (5′-3′)	Amplicon size
Bta-miR-124a	R-T: GTCGTATCCAGTGCAGGGTCCGAGGTATTCGCACTGGATACGACCTTGGC	
	F: CGCGTAAGGCACGCGGTGAAT	
	R: ATCCAGTGCAGGGTCCGAGG	
*U6*	R-T: CGCTTCACGAATTTGCGTGTCAT	
	F: GCTTCGGCAGCACATATACTAAAAT	
	R: CGCTTCACGAATTTGCGTGTCAT	
*PECR*	F: CCAGCAGTGGAGTAAGGATCAATAGTG	181bp
	R: GCAGGAGACAGCAGGAAGCATAC	
*GADPH*	F: CGGCACAGTCAAGGCAGAGAAC	116bp
	R: CCACATACTCAGCACCAGCATCAC	
*ELOVL2*	F: GGCTGCCTCATCTTCCAGTCTTC	169bp
	R: TTACTCCGTTCGCTGTGCTGAAG	

*Note*: The genes in the above table are all from dairy cow species.

## Data Availability

The data used to support the findings of this study are included within the article.
